# Respuestas y resistencias en la lucha contra la viruela, Chile, siglos XVIII-XX

**DOI:** 10.1590/S0104-59702026000100012

**Published:** 2026-06-01

**Authors:** Paula Caffarena

**Affiliations:** i Doctora en Historia, Centro de Investigación y Documentación/Escuela de Historia/Universidad Finis Terrae.Santiago – Chile. pcaffarena@uft.cl

**Keywords:** Viruela, Vacunación, Estado, Resistencia, Chile, Smallpox, Vaccination, State, Resistance, Chile

## Abstract

Este artículo examina las respuestas de las autoridades políticas y sanitarias en Chile frente a las epidemias de viruela, centrándose en las herramientas empleadas (aislamiento, variolización y vacunación) y el fortalecimiento institucional que culminó en la obligatoriedad de la vacunación antivariólica. Sostenemos que estas respuestas reflejan la centralización del poder estatal en salud pública y un proceso de consolidación institucional. Sin embargo, la implementación de estas políticas enfrentó una resistencia pasiva, evidenciada en el bajo número de vacunados durante la primera mitad del siglo XX, lo que revela tensiones entre los objetivos sanitarios estatales y su aceptación social.

## Abstract (en)

This article explores how political and health authorities in Chile responded to smallpox epidemics. It focuses on the methods employed (isolation, variolation, and vaccination) and the institutional strengthening that led to mandatory smallpox vaccination. We argue that these responses reflect the centralization of state power in public health and a process of institutional consolidation. However, the implementation of these policies met with passive resistance, as evidenced by low vaccination rates in the first half of the twentieth century. This reveals tensions between state health objectives and their social acceptance.

Estudios históricos previos han señalado que, tanto en Europa como en Hispanoamérica, el siglo XVIII registró la mayor incidencia de epidemias de viruela. La mayoría de los brotes fueron causados por el virus *Variola mayor*, responsable de una enfermedad grave que afectaba principalmente a los niños y que llegó a provocar la muerte de hasta un 50% de los infectados. Aunque menos frecuentes, también se documentaron casos de viruela menor, una variante menos severa que causaba una erupción leve en la piel y tenía una tasa de mortalidad de apenas el 0,2%. Contraer esta forma de la enfermedad era bien considerado, ya que permitía al paciente superar la infección con síntomas moderados y pocas secuelas, además de adquirir inmunidad de por vida frente a ambas variantes del virus (Williams, 2010).

A lo largo de los siglos XIX y XX, la viruela continuó siendo una de las principales enfermedades infecciosas que afectaron al mundo, causando millones de muertes antes de su erradicación en 1980. La Organización Mundial de la Salud (OMS) estima que durante el siglo XX aproximadamente trescientos millones de personas en todo el mundo murieron a causa de la viruela (Commemorating…, 8 mayo 2020). Su elevada letalidad la convirtió en una preocupación prioritaria para los Estados, que asumieron un papel activo en su control.

A partir de las perspectivas historiográficas que ofrece la historia de la salud pública, que centra su atención en el análisis de las políticas sanitarias, las estrategias de prevención de enfermedades y la participación del Estado (Armus, 2024), este artículo busca analizar las respuestas de las autoridades políticas y sanitarias en Chile frente a las epidemias de viruela, desde fines del siglo XVIII, momento en que éstas se intensificaron, hasta 1950, fecha de la última epidemia de viruela en el país. Esta mirada de largo plazo ofrece una percepción novedosa respecto de investigaciones previas y nos permitirá observar continuidades y cambios en el desarrollo institucional y cómo éste incidió en la implementación y aceptación de la vacunación en diferentes momentos. Buscamos identificar las herramientas sanitarias empleadas y el proceso de fortalecimiento institucional que culminó con la promulgación del decreto de obligatoriedad de la vacunación antivariólica en 1918, así como explorar el comportamiento de la población frente a estas políticas.^1^

A modo de hipótesis, proponemos que la respuesta de las autoridades políticas y sanitarias en Chile frente a las epidemias de viruela, mediante herramientas como el aislamiento, la variolización y la vacunación, reflejó una creciente centralización del poder estatal en el ámbito de la salud pública y un proceso de consolidación institucional que culminó en la obligatoriedad de la vacunación antivariólica. Sin embargo, si bien la vacunación se transformó en la principal herramienta de control sanitario para enfrentar la viruela, persistió una resistencia pasiva de una parte de la población. Esta se reflejó en el bajo número de vacunados durante la primera mitad del siglo XX, lo cual dejó en evidencia las tensiones entre los objetivos de las políticas de salud y su implementación.

La historiografía en torno a las epidemias de viruela y a la vacunación es extensa, por lo que este artículo reconoce y, por tanto, se construye sobre importantes aportes historiográficos previos, los cuales abordan tanto el impacto de las epidemias de viruela en la sociedad como el papel de la vacunación antivariólica en la contención de éstas y su posterior erradicación (Williams, 2010; Warren, 2010; Ramírez, 2022; Hopkins, 2002). En los últimos años, han surgido miradas más críticas sobre este último proceso, pues si bien se reconoce que la erradicación de la viruela es un gran logro sanitario, también se observa que promovió una visión de la salud pública que priorizó el control de enfermedades individuales mediante tecnologías, como son las vacunas, dejando de lado enfoques integrales de salud pública, como son las mejoras de las condiciones socioeconómicas y ambientales, fundamentales para abordar la raíz de muchas enfermedades (Cueto, Palmer, 2015; Birn, 2016; Porras Gallo, Ballester Añón, 2016; Blume, 2024; Carter, 2023; Chávez, Soto, 2018). Del mismo modo, han surgido argumentos en contra de la vacunación basada en la obligatoriedad y en los mecanismos de coacción, en la medida que esta estrategia puede dejar un residuo de resentimiento que deteriore la actitud del público respecto de la siguiente campaña de vacunación (Bhattacharya, 2006).

Para los países de América Latina, la historiografía ha mostrado que la contención de la viruela y la implementación de herramientas sanitarias, entre ellas, la vacunación, conformaron un proceso complejo que reflejó las tensiones entre las políticas de salud y las realidades sociales de la época (Cueto, Palmer, 2015). A lo largo del tiempo, las resistencias sociales y las dificultades institucionales para implementar, por ejemplo, las campañas de vacunación, fueron factores relevantes que las autoridades debieron sortear para conseguir el control de la enfermedad (Di Liscia, 2011, 2021). Es así como se observa que una de las mayores barreras fue la resistencia de la población, la cual se fundó en la falta de información, la desconfianza y en las debilidades que mostraron las instituciones sanitarias en América Latina para difundir la vacuna. Es por ello que la población prefería evitar la vacunación (resistencia pasiva) en lugar de oponerse activamente (Agostoni, 2016). Al respecto, un análisis relevante es el realizado por Needell (1987), que examina la Revolta da Vacina, de 1904, en Rio de Janeiro, Brasil. El autor sostiene que la revuelta no fue simplemente una reacción contra la vacunación obligatoria contra la viruela, sino un fenómeno más amplio que reflejó la tensión entre el proyecto modernizador de las élites y la resistencia popular frente a la exclusión social y la imposición autoritaria de medidas de salubridad.

También el caso chileno cuenta con estudios específicos sobre la viruela, centrados, por una parte, en el impacto de las epidemias y, por otra, en la difusión de la vacunación (Caffarena 2016, 2020, 2022; Caffarena, González, 2023; Dauer, 2020). Los aportes realizados por William Sater (2003) y María Josefina Cabrera (2008) en torno a la obligatoriedad de la vacunación en Chile nos han permitido establecer el peso que tuvieron los factores políticos en dicha discusión.

Con el fin de contextualizar el desarrollo de las principales estrategias sanitarias para el control de la viruela, este artículo se estructura en dos partes. En la primera de ellas se analiza el aislamiento, la inoculación y la vacunación en tanto medidas sanitarias empleadas para contener la viruela y se examina el proceso de fortalecimiento institucional que culminó con el decreto de obligatoriedad de la vacunación antivariólica y, en la segunda, se exploran las formas de resistencia social frente a las herramientas sanitarias utilizadas en la contención de dicha enfermedad.

Considerando el amplio espacio de tiempo que este estudio aborda, se han utilizado fuentes de diversos periodos y procedencia. Para el siglo XVIII, la documentación entre el gobernador y el cabildo, contenida en el Fondo Capitanía General ha sido fundamental. Para el siglo XIX, Ministerio del Interior y los reportes del *Anuario Estadístico* nos han permitido comprender y contrastar los datos entregados. Para el siglo XX, la prensa, la documentación de la Dirección de Sanidad y publicaciones oficiales como las Memorias de la Junta de Vacuna han sido fundamentales en el seguimiento de la política de vacunación.

## Herramientas sanitarias y fortalecimiento institucional en el control de la viruela

Para el territorio que actualmente ocupa Chile, hay registros de epidemias de viruela desde el siglo XVI, sin embargo, fue durante el siglo XVIII que esta enfermedad afectó a Chile de manera recurrente. Es probable que el aumento de los contactos entre continentes hiciera aumentar su frecuencia, lo cual se tradujo en que se tornara endémica en lugares como Inglaterra, España y la mayor parte de Hispanoamérica (Caffarena, 2016).

A lo largo de dicho periodo, los estragos de la enfermedad quedaron plasmados en los testimonios de la época; por ejemplo, a raíz de la epidemia que afectó a Concepción en 1789, los médicos Juan Antonio Ríos y Pedro Manuel Chaparro alertaron a las autoridades locales de que “la ciudad de Concepción estaba amenazada de una cuasi total despoblación” (Capitanía General, 1789, fj.54) si no se ponía freno a la epidemia. Durante el siglo XIX, la viruela continuó afligiendo a la población. Si bien los registros son heterogéneos y discontinuos, lo cual impide ofrecer estadísticas completas a nivel nacional, sabemos que la viruela se presentó de forma epidémica con una alta letalidad. Por ejemplo, entre 1811 y 1812, se desarrolló una epidemia en Santiago, y, de los enfermos que ingresaron al Hospital San Juan de Dios, fallecieron cerca del 26%. Una nueva epidemia afectó a la zona central de Chile en 1830, y, de acuerdo con lo indicado por el médico Thomas Leighton, al Hospital de Valparaíso ingresaron 242 enfermos de viruela, de los cuales falleció el 31%. Durante la segunda mitad del siglo XIX, la población siguió padeciendo epidemias de viruela. La tendencia fue fluctuando con alzas importantes dadas por los brotes epidémicos de 1879, 1885 y 1889 (Dirección General de Estadística, 1910). Entre 1865 y 1874, por ejemplo, fueron atendidos 7.163 enfermos de viruela en el Lazareto de Playa Ancha, y el 31% de ellos falleció (Laval, 2012).

Desde 1920, las epidemias de viruela fueron menos frecuentes y la enfermedad se presentó con casos aislados (Estadística Chile, ene. 1928). Hubo brotes en 1931 y 1935, y luego en 1944 en la Oficina Salitrera Anita en Tarapacá y en 1948 en la Oficina Pedro de Valdivia de Antofagasta. La última epidemia de viruela que se registró en Chile ocurrió en 1950 y fue considerada una epidemia de viruela menor o alastrim, la cual presentó un cuadro clínico benigno, sin complicaciones y con una baja letalidad, del 0,45% (Laval, 2003). En total, se registraron 3.414 casos aproximadamente. En 1959, y gracias al apoyo financiero y ayuda técnica que la Oficina Sanitaria Panamericana entregó, Chile declaró la erradicación de la viruela en el país (Oficina Sanitaria…, 22 oct. 1950).

A partir del desarrollo histórico de la viruela en Chile, es posible identificar dos grandes etapas que reflejan la evolución en el involucramiento de las autoridades políticas y médicas en el control de la enfermedad. En un primer momento, predominaron las iniciativas de carácter reactivo, destinadas a responder a las emergencias sanitarias provocadas por las recurrentes epidemias de viruela. Posteriormente, se observa un giro hacia la sistematización de esfuerzos orientados a establecer una institucionalidad estable para la práctica de la vacunación, que se consolidó como la herramienta central en la lucha contra la enfermedad.

## Aislar, variolizar, vacunar

Una de las particularidades que ofrece estudiar la viruela en un amplio periodo de tiempo, es que nos permite observar el papel de las autoridades y los mecanismos de control que se recomendaron para la contención de la enfermedad. Si bien hubo tratamientos médicos que se aplicaron de manera transversal a lo largo de los siglos XVIII y XIX, como sangrías, terapias frías, cálidas o purificación del aire, fue la implementación de cuarentenas, la inoculación o variolización, y posteriormente la vacunación, lo que se tradujo en un involucramiento más estrecho de las autoridades. Frente a la alta letalidad de la viruela, éstas debieron recomendar o no la implementación de ciertas medidas e idear mecanismos que permitieran su ejecución, posicionando la discusión en la esfera pública. Si bien aislar, variolizar y vacunar fueron herramientas sanitarias distintas, estuvieron vinculadas. En los tres casos, se necesitó de la autoridad política para regular su uso y ejecución y de la autoridad sanitaria para validarlas.

El nivel de involucramiento de la autoridad para decretar una cuarentena y poner en marcha la inoculación, podemos observarlo en el caso de la epidemia que afectó a Concepción en 1789, momento en que la decisión de aislar a los enfermos de las personas sanas y de recomendar la variolización de la población fueron temas centrales durante las reuniones que sostuvo el cabildo de esa ciudad a raíz de la epidemia. Se hizo un llamado a sacar a los enfermos fuera de la ciudad para su curación y a instaurar estrictas medidas de control para impedir la comunicación con los lugares afectados de viruela. Así, se propuso la “extracción de los que resulten con la peste, multiplicando los celadores por varias calles o cuarteles, para que aquellos no se oculten y tomando las providencias coactivas que su celo estime conducentes a mismo fin” (Indiferente…, sep. 1789, fj.577). En otras palabras, la necesidad de aislar a los contagiados debía ir más allá de la voluntad individual, imponiendo multas e incluso penas de cárcel para quienes no cumplieran con lo decretado por las autoridades.

Un comportamiento diferente se observa cuando las autoridades debieron pronunciarse respecto a la inoculación, otra de las herramientas que se utilizó para el control de la epidemia de viruela de 1789 y que, de acuerdo con el médico español Francisco Gil (1784, p.30), “se trataba de comunicar la materia variolosa de unos en otros con el fin de conseguir inmunidad”. Si bien la variolización era una técnica muy antigua, durante la segunda mitad registró un impulso que estuvo dado por su aceptación y difusión en el mundo occidental. A diferencia de la separación de enfermos, la inoculación era una práctica que tenía un grado mayor de controversia, por lo que la autoridad no forzó su uso ni discutió medidas coercitivas para su implementación, sino que se limitó a autorizarla y a regular el ejercicio de ella. En la ciudad de Concepción, por ejemplo, el gobernador, Ambrosio O’Higgins, envió al inoculador Juan José Morales, ya que solo las personas certificadas podían practicarla.

Todo ello muestra que, para el caso que presentamos, las autoridades políticas y médicas actuaron de manera reactiva frente a la epidemia y gestionaron la crisis sanitaria a partir de las dos herramientas que tenía disponibles: el aislamiento y la variolización. La variolización fue un medio recomendado para contener la viruela y, por tanto, fue puesto a disposición de la población, sin fijar costos ni condiciones especiales para el inoculado. En el caso de la separación de contagiados, se establecieron sanciones, como penas de cárcel y multas, para quienes no siguieran las indicaciones dadas.

La llegada de la vacuna contra la viruela a Chile, a fines de septiembre de 1805, marcó un hito importante, pues los estragos causados por la viruela podían ser detenidos si se lograba inmunizar a la población a través de la vacunación. En este sentido, difundir la vacuna se transformó en una preocupación para las autoridades políticas y médicas que asumieron que la difusión de ella era parte de sus atribuciones.

El 5 de julio de 1805 llegó a Montevideo un comerciante portugués, proveniente de Rio de Janeiro, que transportaba consigo esclavos negros, entre los más pequeños de ellos traía la vacuna conservada de brazo a brazo. Las autoridades del periodo rápidamente tomaron muestras del fluido y, al mes siguiente, se envió la vacuna a las provincias de Salta y Córdoba, así como a Chile y a Lima; allí fue recibida por las autoridades de dichos territorios. A fines de septiembre de 1805, el gobernador de Chile recibió el fluido vacuno y autorizó a Pedro Manuel Chaparro para que realizara las primeras vacunaciones (Indiferente General, ago. 1805, fj.1292).

A pesar de las innumerables dificultades políticas, económicas y sociales, producto de las guerras de independencia, las autoridades políticas de la época comprendieron la importancia de la vacuna para frenar el avance de la viruela e intentaron institucionalizar la vacunación a través de la formación de la Junta de Vacuna de 1812 y de la Junta de Sanidad de 1822. Adicionalmente, otros actores se involucraron también en la tarea. Por ejemplo, la Iglesia cumplió una función importante en las estrategias y medidas dispuestas para propagar la vacuna. No sólo se trató de que los curas mencionaran la disponibilidad del fluido en sus sermones, sino de que éstos anunciaran públicamente los días y lugares establecidos para vacunar y persuadieran a los fieles de acudir a ellos. Además, se pidió que después de la misa los curas publicaran en la puerta de la Iglesia, “el tiempo en que el vacunador ha de operar allí, y promuevan todos los medios para que se estimulen a vacunarse cuantos lo necesiten, y se obligue a los morosos” (Fernández de Leiva…, 1808, fj.3). Al papel persuasivo de la Iglesia se sumaron los mecanismos coercitivos que el incipiente Estado implementó. Por ejemplo, mandó a reclutar personas en las ceremonias religiosas, entre quienes concurrían a las plazas o a cualquier tipo de celebración, en la cárcel y también en las casas de recogidas, “valiéndose hasta de la fuerza, con auxilio de alguaciles, o de las guardias militares próximas” (Vacuna, 23 abr. 1812, p.46).

A pesar del impulso institucional, de las sanciones que se anunciaban y de los mecanismos de persuasión que se aplicaban, durante los primeros 30 años del siglo XIX, la propagación de la vacuna no pasó de ser una iniciativa individual, esporádica y a cargo de los vacunadores que contaban con fluido de buena calidad, lo cual se vio reflejado en el número de vacunados que no sobrepasó las diez mil personas, número muy baja como para contener la aparición de una epidemia de viruela.

## Del fortalecimiento institucional al decreto de obligatoriedad de la vacunación antivariólica

**Figura 1:**
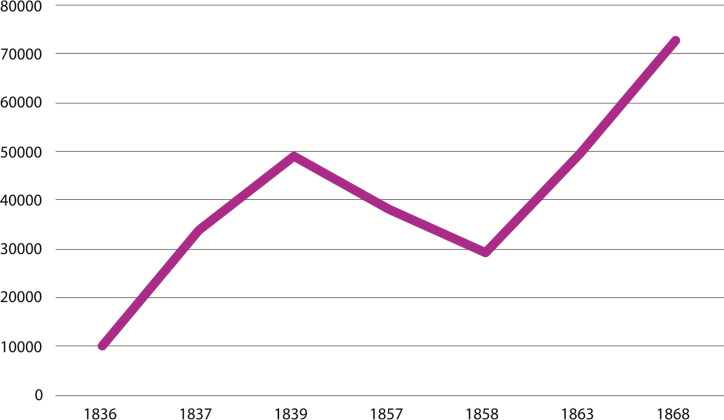
: Número total de vacunados entre 1836 y 1868 (Fuente: elaboración propia a partir de documentación proveniente del Ministerio del Interior, 1869)

Un cambio importante se produjo con la creación de la Junta de Vacuna en 1830, que dotó a la vacunación de una institucionalidad más estable, en el marco de un Estado nacional que presentaba fuertes rasgos de centralización. Esta junta tuvo su propio reglamento, el cual fue promulgado el 4 de julio de 1830 y estableció que la vacunación dependía de la Junta Central de Vacuna que, a su vez, estaba supeditado al Ministerio del Interior. Asimismo, existían juntas particulares establecidas en los distintos departamentos de la República, que llevaban la vacunación más allá de la capital (Caffarena, González, 2023). Su creación se tradujo en cambios en la política de vacunación, que, si bien mantenía continuidad con lo hecho durante la década de 1820, introdujo nuevos elementos en la política, dándole a esta un carácter coercitivo que antes no había tenido. Los vacunadores, por ejemplo, fueron sometidos a un mayor control, a fin de aumentar el número de vacunados a lo largo de todo el territorio. Debieron acrecentar la cantidad de vacunaciones que debían realizar y se incrementaron las sanciones que se aplicarían si no cumplían con su deber. El objetivo fue expandir la cobertura y hacer más eficiente el trabajo (Caffarena, 2020). Como muestra la Figura 1, a contar de 1830, el número de vacunaciones totales aumentó considerablemente con respecto al periodo anterior. En este sentido, el fortalecimiento institucional y la centralización estatal permitieron una mejor difusión de la vacunación a lo largo de todo el territorio.

La lucha por controlar la viruela y por inmunizar masivamente a la población se mantuvo durante todo el siglo XIX, pues si bien la institucionalidad creada en torno a la vacunación permitió aumentar el número de vacunados, aún el porcentaje de éstos con respecto al total de la población era insuficiente para detener el avance de la viruela. Es así como a fines del siglo XIX se observa un cambio en cómo las autoridades abordaron el control de la viruela, pues ya no solo se trató de dotar de institucionalidad a la práctica ni de ponerla a disposición de la población, sino de hacer de ella algo obligatorio.

El primer proyecto que se presentó con el fin de decretar la vacunación antivariólica obligatoria fue el de Ramón Allende Padín, en 1877. El proyecto se llamó “Jeneralización de la vacuna”, y si bien logró ser discutido en el Congreso en 1882, no consiguió su aprobación. De acuerdo a las investigaciones de Josefina Cabrera (2008) y William Sater (2003), los legisladores no vetaron el proyecto de Allende Padín porque consideraban que la vacunación era una charlatanería. Por el contrario, la mayoría de ellos habían sido vacunados. La cámara baja rechazó la ley que exigía la vacunación obligatoria por razones políticas. Por una parte, los diputados creían que le daba al Poder Ejecutivo la capacidad de manipular el proceso político. Así lo resumió el diputado Ricardo Letelier (13 jul. 1882, p.209), cuando señaló que “los estragos de la viruela no son nada en comparación de los que puede producir esa otra plaga que se designa con el nombre de autoritarismo”. Por otra, la discusión en torno a la preeminencia de la libertad individual frente al bien común también se expresó en los argumentos expuestos en los debates legislativos. Por ejemplo, el diputado Jordán (13 jul. 1882, p.233) expresó:

El Proyecto del Senado es un ataque directo contra la libertad individual… Tengo la convicción de que todos los Honorables Diputados que sostienen la teoría de la vacunación forzosa se encuentran animados de las mejores intenciones; pero me permito preguntarles si han pensado también qué es lo que encierra este proyecto arbitrario. Nada menos que una intervención, un atropello al derecho santo de la libertad individual.

A diferencia de estas opiniones, el doctor Adolfo Murillo (1882, p.2035) expresó que “veo a los países más libres de Europa, como son la Inglaterra y la Suiza, orgullosos de su libertad, adoptar la vacunación obligatoria… Sostengo que nadie tiene derecho para ser un foco de infección que perjudique al vecino, y que la autoridad debe velar por el derecho de terceros”.

También fue tema de debate la capacidad del Estado para hacer de la vacunación una práctica obligatoria. A través de las discusiones parlamentarias, se observa que la difusión de la vacuna enfrentó una serie de obstáculos de orden práctico e institucional, tales como el mal funcionamiento de la Junta de Vacuna, a la cual se le reprochaba la escasez e ineficiencia de los vacunadores, la falta del fluido o la mala calidad de éste e incluso la transmisión de enfermedades. Por tanto, el problema de la masificación de la vacunación también se debía a la debilidad institucional, lo cual ponía en duda la pertinencia de decretar la obligatoriedad. El diputado Letelier (13 jul. 1882, p.209), por ejemplo, se preguntaba, “y si no hemos tratado de procurar este elemento de salvación, ¿por qué hemos de ocurrir a la restricción de la libertad individual?”.

Aunque esta primera propuesta fue rechazada por el Congreso, nuevas normas asociadas a la vacunación se impusieron. Una de las más relevantes fue la exigencia del certificado de vacunación para el ingreso a la escuela (Ponce, 1885), medida que se decretó incluso antes de la aprobación, en 1887, del decreto del presidente Balmaceda, que promulgó la vacunación obligatoria para los recién nacidos vivos. Si bien esta exigencia no se tradujo en que todos los niños en edad escolar estuvieran vacunados, la relación entre vacuna y educación se constituyó en una de las herramientas que el Estado utilizó para forzar a la población a recibir la vacunación. A lo largo del siglo XX, el certificado de vacunación para acceder a la educación se mantuvo y en casos de epidemia se reforzó. Así ocurrió en la epidemia de 1950, donde no solo se exigió dicho certificado, sino que también se llevó la vacuna a las mismas escuelas y se impusieron certificados de vacunación para viajar, para acceder a los servicios públicos y a instituciones particulares y privadas (Exigen…, 2 mar. 1952, p.18). Todo ello con el fin de ampliar la cobertura de la vacuna en la población más susceptible de contraer la enfermedad.

La promulgación del Código Sanitario en 1918 fue otro hito importante en la difusión de la vacuna antivariólica. Por una parte, incorporó cambios en la institucionalidad, creando el Consejo Superior de Higiene y la Dirección de Sanidad, correspondiéndole a esta última dirigir los servicios de vacunación. Por otra, estableció la obligatoriedad de la vacunación antivariólica para todos los habitantes de Chile.

La vacuna, estipulaba el Código Sanitario en su artículo 57, debía ser puesta dentro del primer año, a contar desde el día en que empiece a regir este Código a todas las personas que en esa fecha no hubieren sido vacunadas o revacunadas, respectivamente. Luego, debían ser revacunados en el décimo y en el vigésimo respectivamente. Se estableció que la vacunación sería gratuita, y que se practicaría a domicilio o en los locales designados por la Dirección General. Tal como ocurrió con otras medidas, el Código también estableció sanciones. Se contempló, por ejemplo, la aplicación de una multa “de diez a cincuenta pesos, sin perjuicio de la vacunación o revacunación” (Código Sanitario..., 1918, p.23). Este código estuvo vigente hasta 1931, momento en que se promulgó un segundo Código Sanitario, el cual conservó la obligatoriedad de la vacunación en los mismos términos que lo había hecho el Código de 1918. Para la década del 1930, la institucionalidad sanitaria se había robustecido y centralizado a través de la creación, en 1924, del Ministerio de Higiene, Asistencia y Previsión Social. La vacunación, por su parte, desde 1927 dependió de la Oficina de Inmunización Antivariólica, que estaba supeditado a la Dirección General de Sanidad.

A pesar de los decretos, reglamentos y sanciones propuestas, la implementación de la obligatoriedad no resultó sencilla, pues exigió un permanente control por parte de la autoridad sanitaria que fue difícil de implementar. La última epidemia de viruela que se registró en Chile en 1950 reveló que aún había un número importante de la población que no estaba inmunizada, y, tal como reconoció el ministro de salud, Jorge Mardones Restat, “si la ley de vacunación obligatoria se hubiese cumplido como debe cumplirse, la epidemia de alastrim no se habría producido” (No habría…, 18 abr. 1950, p.28).

Entre 1900 y 1940, el total de vacunaciones anuales no superó las seiscientas mil personas. Así, en 1927, por ejemplo, el número de vacunados solo alcanzaba el 13% de la población total, según informó la Oficina de Inmunización Antivariólica (Estadística Chile, ene. 1928). Es importante considerar que existían diferencias regionales importantes. Por ejemplo, en Tarapacá se vacunó en 1927 a un 74% de la población, mientras que en Chiloé solo lo hizo un 3,8%. Estas diferencias se explicaban, de acuerdo al reporte de la Oficina de Inmunización, porque en las provincias del Norte la población vive concentrada en ciudades y oficinas salitreras, mientras que en el Sur se encuentra dispersa en el campo, lo cual dificulta las labores de la autoridad sanitaria (Estadística Chile, ene. 1928).

La implementación de la obligatoriedad de la vacunación en 1918 no se tradujo en un aumento sostenido del número de vacunaciones, por lo que siempre existió un grupo de población susceptible de contraer viruela. No está del todo claro a qué se debió esto. Puede haber influido el hecho de que el decreto de vacunación obligatoria no se aplicó de manera estricta, lo que dejó la inmunización contra la viruela en manos de la voluntad individual. También pudieron influir la falta de infraestructura sanitaria necesaria para implementar la medida, así como las resistencias sociales hacia la práctica de la vacunación. Otra posibilidad es que las epidemias hubiesen disminuido y, con ello, la percepción de riesgo asociada a la viruela.

Lo que sí se puede observar es que la intensificación de la vacunación tendió a coincidir con el surgimiento de brotes epidémicos. En esos contextos, aumentaba la percepción de riesgo en la población y se aplicaba con mayor rigurosidad la ley de obligatoriedad de la vacunación y sus sanciones.

Así ocurrió en 1903, cuando una epidemia de viruela afectó de manera importante a la ciudad de Santiago y el presidente de la Junta de Vacuna reconoció que habían tenido que implementar un servicio extraordinario de vacunación, que tuvo que ser reforzado a medida que los enfermos aumentaban (Memoria…, 1905, p.6). Una situación similar ocurrió en 1948, cuando una niña de 3 meses que era parte de una familia que ya había tenido viruela, contrajo la enfermedad. Ante ello, el director de Sanidad, Nacianceno Romero, determinó que toda la población que habitaba la oficina fuera vacunada contra la viruela, así como el establecimiento de cordones sanitarios, a fin de evitar la propagación de la enfermedad (Un nuevo…, 8 may 1948). Lo mismo ocurrió para la epidemia de 1950, que logró ser controlada gracias a un estricto programa de vacunación que se puso en marcha y que, en los primeros diez días, logró inmunizar a 1.327.400 personas, de las cuales 890 mil correspondieron a Santiago. Para el 9 de agosto de 1950, el total de vacunados ascendía a 5.328.307, cifra cercana al 90% de la población del país en esa época (Laval, 2003). En esta epidemia se estableció y difundió a través de la prensa que se multaría con cincuenta pesos a las personas que fueran sorprendidas sin vacunarse, multa que se aumentará a mil pesos en casos de reincidencia. Adicionalmente, la policía tenía la facultad de “llevar a aquellos no inoculados hasta la posta de vacunación más cercana, sin perjuicios de las multas señaladas” (La vacunación…, 13 abr. 1950, p.41).

En 1957 también se realizó un alto número de vacunaciones, las cuales se desarrollaron en las provincias limítrofes con Bolivia y Argentina. Se trató de un programa extraordinario que se llevó a cabo con el objeto de evitar la propagación a Chille del brote de viruela que se presentó en Bolivia. Durante 1959, a raíz de un único caso confirmado de viruela en Antofagasta se practicó un número de vacunaciones totales a nivel nacional, cercano a 3.000.000” (Servicio Nacional de Salud, 1959, p.47).

**Tabla 1:** : Total anual de inmunización antivariólica, 1950-1959 (incluye primera dosis, segunda dosis y revacunaciones)

Año	Vacunaciones
1950	5.328.307
1951	321.508
1952	634.735
1953	798.257
1954	498.234
1955	605.704
1956	783.188
1957	1.273.506
1958	682.325
1959	3.000.000

Fuente: Servicio Nacional de Salud (1959)

## Formas de resistencia social en la lucha contra la viruela

Pese al riesgo que representaba la viruela, la población no siempre siguió las medidas establecidas por las autoridades. A lo largo del siglo XVIII, por ejemplo, la implementación de cuarentenas generó cierto grado de resistencia por parte de los enfermos y sus familias, quienes en ocasiones se negaron a abandonar sus hogares o a salir de la ciudad, optando por ocultar a los variolosos y evitar el llamado a los médicos para su atención (Indiferente…, sep. 1789, fj.587). Un caso que ejemplifica esta situación se registró durante la epidemia de Concepción en 1789, cuando el intendente de la ciudad, Francisco de la Mata Linares, informó al gobernador Ambrosio O’Higgins que no fue posible aplicar las medidas de cuarentena más estrictas. Por un lado, resultó imposible contabilizar con precisión los casos de viruela; por otro, la propia población mostró resistencia al aislamiento (fj.587).

La recomendación de practicar la inoculación en la epidemia que afectó a dicha ciudad también fue resistida por la población, lo cual estuvo vinculado con la incertidumbre que generaban los nuevos tratamientos, así como con el desconocimiento de los efectos de inocular y con el temor al contagio de viruela que esta práctica podía provocar. Diego Barros Arana (2002) ha planteado que, si bien fue una medida preventiva útil, fue aceptada con muchas resistencias y que se difundió mayormente entre las clases acomodadas de las ciudades con mayor cantidad de habitantes. En el campo y en los pueblos más pequeños fue muy difícil de aplicar, ya fuese por falta de medios o de personas que supieran hacerlo. Además, “las gentes se resistían tenazmente a someterse a ella, persuadidas de su ineficacia, o de que era a veces origen de gravísimas enfermedades” (p.270). Frente a ello, las autoridades buscaron persuadir a la población del beneficio de la práctica, por ejemplo, a través del efecto ejemplificador que se generaba cuando circulaba la noticia de que algún habitante de la ciudad con cierta notoriedad social era inoculado (Capitanía General, 1790, p.105-108).

Estos ejemplos muestran que en el caso de la inoculación, el desconocimiento y la desconfianza hacia una práctica médica nueva constituyeron un obstáculo significativo para su difusión. En cuanto a las medidas de aislamiento, la separación de los enfermos de sus familias fue objeto de una fuerte resistencia por una parte de la población, probablemente, debido a la incertidumbre respecto a las condiciones de los lugares de aislamiento y a la alta probabilidad de no volver a ver a sus familiares. En este contexto, la aplicación de sanciones (multas o penas de cárcel) y fiscalización de la autoridad para el cumplimiento de las disposiciones tuvieron un alcance limitado.

Los primeros intentos de difusión de la vacuna antivariólica también debieron sortear dificultades. Por una parte, la situación bélica en que se encontraba el territorio, producto de las guerras de independencia, repercutió directamente en la afluencia de personas a los vacunatorios, pues, al menos en Santiago, si bien existió un vacunatorio, el problema estuvo en que las personas no acudieron al lugar, ya que existía el temor de que fuesen tomados para la guerra cuando concurrían a recibir la vacuna (Capitanía General, jun. 1814). Por otra parte, la resistencia de la población a recibir la vacuna fue un fenómeno persistente, motivado tanto por la desconfianza en su efectividad como por el temor a posibles efectos adversos. Estos temores no eran completamente infundados. Una situación frecuente en la época era la denominada “falsa vacuna”, que se producía cuando se inoculaba a una persona con fluido en mal estado, lo que impedía generar inmunidad efectiva, aunque el individuo creyera estar protegido (Caffarena, 2016).

Desde fines del XIX, con el desarrollo de una institucionalidad específica para la vacunación y gracias a mejoras técnicas en el tipo de fluido que se utilizaba, la vacunación comenzó a ser un procedimiento más seguro y confiable para la población.^2^ Sin embargo, no por ello desaparecieron las resistencias a la práctica.

El bajo número de personas que concurrían a recibirla motivó a las autoridades a pedir apoyo a quienes mantenían un poder persuasivo en la población. De eso dio cuenta el presidente de la Junta Central de Vacuna en la memoria presentada en 1904, donde explicó que para aumentar la cobertura de la vacunación se había pedido “su eficaz cooperación al señor Arzobispo de Santiago, al señor Alcalde, al señor Inspector General de Instrucción Primaria cerca de las numerosas escuelas de su dependencia” (Memoria…, 1905, p.13). Esta solicitud fue bien recibida por el arzobispo, quien ofreció que “en las instrucciones dominicales o en la forma que Ud. juzgue más oportuna, se empeñe en persuadir a sus feligreses de la conveniencia de que acudan a vacunarse sin esperar a que el contagio sea inminente” (p.15).

Estos testimonios muestran que, a pesar de los procesos de centralización estatal, fortalecimiento institucional y de la aprobación de la obligatoriedad de la vacunación, la población aún no acudía masivamente a vacunarse. Por el contrario, se observa que justamente en el periodo en que se implementan políticas coercitivas, como las multas por no vacunarse o el requerimiento del certificado de vacunación como requisito para ingresar a la escuela, surge una resistencia más activa, la cual, si bien no se expresó en la organización de movimientos, manifestaciones o protestas, sí se tradujo en la circulación de impresos que cuestionaron la eficacia y benignidad de la vacunación.

El autor de estas publicaciones fue Alfredo Helsby (1862-1933), quien cuestionó los reportes oficiales que respaldaban la eficacia de la vacuna y su relación con la disminución de casos de viruela. Alertó sobre los efectos nocivos que la vacuna podía tener en los seres humanos, ya fuese de manera inmediata o a largo plazo y sobre la posibilidad de que ésta fuera la causante de propagar enfermedades o incluso la causa de éstas. También discutió el papel del Estado en materia sanitaria, rechazando las medidas de control que se estaban aplicando frente a quienes decidían no vacunarse, tildándolas de autoritarias. Uno de los puntos que refutó, por ejemplo, fue la prohibición de que los niños no vacunados asistieran a las escuelas públicas, medida que para Helsby (1898) era arbitraria y atentaba contra los derechos fundamentales de los individuos.

Lo anterior sugiere que en Chile hubo resistencia a la vacunación, la cual si bien fue en su mayoría pasiva (Cabrera, 2008), tuvo una expresión activa acotada a partir de las publicaciones de Helsby. La resistencia pasiva se expresó en que las personas no acudieron a los vacunatorios o se negaron a recibir la vacuna, alegando falta de tiempo o información.

Si bien la documentación no nos permite explorar con profundidad todos los factores que incidieron en ello, a partir de 1930, los reportes oficiales, dieron cuenta de los casos de resistencia. Por ejemplo, en 1935, el vacunador Belarmino González envió al director general de sanidad, un reporte de las personas que habían mostrado resistencia a vacunarse (Dirección General de Sanidad, 1931). Dicha notificación era también enviada al residente de la casa a la que el vacunador había acudido, para notificarle la obligación que tenía de presentarse en el vacunatorio al día siguiente, con los niños que no habían sido vacunados o revacunados. Esto, “bajo apercibimiento de multa de cien mil pesos en conformidad a lo establecido en el art. 230 del Código Sanitario, sin perjuicio de disponerse la vacunación o revacunación antivariólica con el auxilio de la fuerza pública” (Dirección General de Sanidad, 1931).

En general los informes no presentan mayores detalles ni dan cuenta de si finalmente los niños fueron llevados al vacunatorio. Una excepción la encontramos en 1935, momento en que se presentó un caso importante de resistencia a la vacunación que derivó en la aplicación de una multa que, posteriormente, fue apelada. Todo este proceso dejó un registro que reveló tanto el procedimiento que las autoridades seguían en estos casos, como las razones que tuvo un comerciante de Santiago, para negarse a recibir la vacuna.

El dueño de una juguetería de apellido Lama, ubicada en el centro de Santiago, fue acusado de rechazar la vacuna, tanto para él como para el personal de su tienda. Por este motivo, se le cursó una multa de mil pesos a él y de veinte pesos a Ana Lara y Olga Céspedes, trabajadoras del lugar. Los sancionados apelaron a la infracción, indicando que nunca se resistieron a ser vacunados, sino que los vacunadores visitaron la juguetería “en el mes de diciembre, en horas que estaba lleno de público que compraba juguetes para la festividad de Pascua y, en consecuencia, se limitaron a solicitar de los vacunadores que volvieran a hora más apropiada permitiendo, entre tanto, la vacunación de los repartidores que estaban desocupados” (Dirección General de Sanidad, 1936).

A pesar de lo anterior, los informes del Departamento de Inmunización indicaron que la tienda venía siendo visitada desde agosto y que siempre se encontraron con la oposición de su dueño. Sin embargo, una vez que Lama supo de la multa, se apresuró y solicitó un vacunador a domicilio. Finalmente, el comerciante fue vacunado y pidió que se cambiara la multa por una amonestación, alegando que ni él ni sus trabajadoras habían cometido previamente alguna otra infracción. A pesar de los reclamos, la Dirección de Sanidad no accedió a la solicitud (Dirección General de Sanidad, 1936).

Aunque en sus declaraciones Lama no expresó de manera explícita su negativa a ser vacunado, atribuyendo su falta a impedimentos circunstanciales, las autoridades sanitarias observaron en él una actitud hostil hacia el personal encargado de exigir el cumplimiento de la medida. Por esta razón, el inspector general de la Oficina de Inmunizaciones, José Figueroa, solicitó “la aplicación de la multa máxima a este Sr., para el cual había sido inútil todo procedimiento educativo y persuasivo” (Dirección General de Sanidad, 1936).

Esta resistencia se comprende mejor cuando, en los informes finales, Lama expresó que su descontento con el proceder de la autoridad sanitaria se debía a la desconfianza hacia la Dirección General de Sanidad por el fracaso de la campaña de inmunización que buscaba proteger a la población del tifus exantemático (Dirección General de Sanidad, 1936). Para esta campaña se utilizó la vacuna Blanc que aún se encontraba en fase experimental. Sin el seguimiento y control adecuado, de las 550 personas vacunadas, 227 enfermaron de tifus y cinco de ellas murieron (Instituto de Salud Pública, 2008).

La escasez de testimonios directos dificulta determinar con precisión el impacto que pudo tener el episodio relacionado con la vacuna Blanc en la disposición de la población a recibir la vacuna antivariólica. No obstante, al considerar la resistencia a la vacunación vinculada a la desconfianza en la eficacia y seguridad, es posible advertir que se trató de un fenómeno persistente en el tiempo. Desde la introducción de la vacuna en Chile a comienzos del siglo XIX, las autoridades sanitarias ya identificaban factores como la existencia de la denominada “falsa vacuna” como una causa de resistencia entre la población. Durante ese siglo, especialmente en el periodo en que se utilizó la vacuna humanizada, también se registraron casos de transmisión de otras enfermedades a través del procedimiento, lo que afectó negativamente la confianza pública. Si bien en el siglo XX los avances técnicos permitieron disponer de una vacuna antivariólica más segura, episodios como el de la vacuna Blanc pudieron contribuir a la persistencia de ciertas desconfianzas en sectores de la población.

La resistencia pasiva de una parte de la población fue constante y solo se atenuó cuando la aparición de algún brote epidémico aumentó la percepción de riesgo. En este marco, no fue el decreto de obligatoriedad lo que permitió una mayor cobertura en la vacunación, sino más bien las campañas específicas de inmunización implementadas en respuesta a dichos brotes, las que lograron incrementar progresivamente la proporción de personas inmunizadas. Tal como lo señaló el director del Servicio Nacional de Salud en 1958, en Chile la única estrategia efectiva para incentivar la vacunación consistía “en que las autoridades llamaran la atención sobre el peligro potencial de la viruela. En Chile, lo único que hacía reaccionar a la gente era el miedo” (Servicio Nacional de Salud, 15 jul. 1959, p.1).

## Consideraciones finales

Si bien la vacuna constituyó la herramienta sanitaria y tecnológica decisiva para el control y posterior erradicación de la viruela, desde una perspectiva de largo plazo es necesario considerar también el papel que desempeñaron otras estrategias, como las medidas de aislamiento y la práctica de la variolización, en el manejo de la enfermedad. Estas respuestas tempranas a las epidemias sentaron las bases para la construcción de una institucionalidad sanitaria que, con el tiempo, favorecería la adopción de políticas más sistemáticas de control. En el caso chileno, la reacción frente a los brotes de viruela y el proceso de centralización del aparato estatal en salud pública se reforzaron mutuamente: las epidemias presionaron hacia una mayor institucionalización, mientras que la progresiva centralización estatal dotó de mayor eficacia y alcance a las respuestas sanitarias. Este proceso permitió la implementación progresiva de políticas de vacunación obligatoria y contribuyó a consolidar el papel del Estado como actor principal en la gestión sanitaria. Sin embargo, esta centralización no se desarrolló de manera lineal ni estuvo exenta de limitaciones, ya que su consolidación enfrentó importantes obstáculos, especialmente relacionados con la debilidad estructural del Estado en términos de infraestructura y disponibilidad de recursos.

En el caso de Chile, la centralización sanitaria fue impulsada significativamente por la creación del Ministerio de Higiene en 1924, que centralizó funciones dispersas y profesionalizó el sector salud, y por el desarrollo de la medicina social, que promovió enfoques integrales y fomentó políticas para reducir inequidades sanitarias. La creación del Servicio Nacional de Salud consolidó los servicios de salud bajo una administración unificada, facilitando la gestión eficiente de recursos y la extensión de servicios a áreas remotas. Además, la colaboración con entidades internacionales como la OMS y la Organización de las Naciones Unidas para la Educación, la Ciencia y la Cultura permitió la adopción de estándares globales y fortaleció las políticas de salud mediante apoyo técnico y económico, asegurando la implementación uniforme y efectiva de programas de salud pública, incluidas las campañas de vacunación.

La resistencia pasiva de una parte de la población a la vacunación contra la viruela contribuyó en las bajas tasas de vacunación en Chile, pero no fue el único factor. La escasez de vacunas y la falta de vacunadores capacitados, especialmente en regiones remotas y rurales, también jugaron un papel crucial. Estas deficiencias logísticas y de recursos limitaron la capacidad del sistema para implementar una cobertura de vacunación eficaz y amplia, lo que contribuyó a las bajas tasas de inmunización observadas durante la primera mitad del siglo XX.

## Data Availability

No están en repositorio.

## NOTAS

1 Si bien la noción de herramienta sanitaria no posee una definición universalmente establecida, en este artículo, nos guiamos por lo indicado por George Rosen (1993, p.106-166), en cuanto se utiliza para describir diversos instrumentos, tecnologías y métodos empleados para prevenir, diagnosticar, tratar o gestionar condiciones de salud y enfermedad.

2 Desde 1888, comenzó a utilizarse en Chile la denominada “vacuna animal”, la cual poco a poco fue reemplazando a la vacuna humanizada o de brazo a brazo. La vacuna animal era preparada en el Instituto Animal ubicado en la Quinta Normal de Agricultura. El fluido se conservaba en glicerina para evitar el contagio con otras enfermedades. Posteriormente, se guardaba en placas de vidrio.
